# Self-Help in Institutional Economy

**Published:** 1913-03-01

**Authors:** 


### SELF-HELP IN INSTITUTIONAL ECONOMY.

### VI.—The Information Bureau.

One of the main reasons for the overlapping which is so frequently complained of in our hospital and Poor-Law system is the want of reliable and authoritative information concerning the capacity of various institutions to deal with special cases. The same reason explains the unfortunate instances which the public designate "hospital scandals," in which a patient is sent from one institution to another, often when his or her condition is liable to be aggravated by such transport, in the vain attempt to find a bed. There are certain times when a hospital is overcrowded; that is to say, when its emergency beds are filled, so that it is impossible for it to admit a single in-patient without unjustifiably infringing its elementary principles of administration. The same thing occurs in Poor-Law infirmaries and in fever hospitals. In such cases the superintendents know that the institutional beds are all occupied; the house surgeons and house physicians know it; the porters, very likely, know it as well. But the public and the public authorities outside the hospital do not know it. An accident happens in a street close to a hospital and the police naturally take the injured person to that hospital, presuming with the usual light-hearted optimism that the public manifests in questions of hospital politics that there is "plenty of room" to take the poor fellow in if it is a case for in-patient treatment. At the casualty department the gravity of the case is recognised; house surgeon confers with house surgeon; there is much gesticulation, much rearrangement of schedules for the take-in week, much scratching of heads, some strong language expressed, and finally the inevitable conclusion that "We haven't got a single bed to spare, officer; you'd better try —— Hospital." Then —— Hospital is 'phoned to and reluctantly acknowledges that it, too, has not got a vacant bed. Sometimes even the preliminary courtesy of 'phoning to the other hospital is omitted, and the constables remove their charge to that hospital only to find that they have made their journey in vain.

They are beginning to deal with these things in a more common-sense fashion elsewhere by means of a system of co-operation which enables every hospital in a certain district to know at a glance what beds are available in all the other institutions within that district. In some parts, notably in Philadelphia in America, at Lyons, and in Vienna, this system is still in its infancy. There it is limited in application and therefore in usefulness, but it serves as a beginning of a policy which is eminently desirable. In Berlin and Hamburg, however, it has progressed considerably, so that to-day the "Bureau of Information" in these cities is an organisation that does much useful work on behalf of the hospitals.

In those countries where the bureau is still in its infancy a number of hospitals have combined to place their daily returns at the disposal of (*a*) the police and (*b*) the local ambulance stations. The arrangement is entirely a voluntary one and is not generally recognised, so that only the returns of a certain number of institutions are available for immediate reference. Limited, however, as the scheme is, it has proved of such great usefulness that its more general adoption has been earnestly advocated. At Berlin the Hospital Information Bureau is a municipal institution, since all the great hospitals are rate supported. It is centrally located and is in telephonic communication not only with all the hospitals, but also with all the various ambulance stations which are spread round the city at the street corners and in the squares of every district. Every morning the superintendents of the various hospitals make up a return, giving details of the beds that are vacant in their institutions. This is posted in the bureau, and as each bed is filled in the course of the day the necessary alterations are announced by telephone and are entered on the official lists at the bureau, so that the clerk in charge is able to tell at a glance whether there is a vacant bed for a particular case at any hospital in any district. Usually particulars of all the vacant beds are given, but the official requirements are that the number of vacant accident beds should be known. When a policeman comes across an accident case in the street he takes it to the nearest ambulance station where first aid is given; the casualty officer in charge, if he judges that the case is severe enough to make in-patient treatment necessary, at once telephones to the bureau to ask if there is a vacant bed for, say, a case of fractured thigh in the nearest hospital in that district. The clerk at the bureau looks at the official list and notes that the district hospital has no vacancies for fractured thigh, but that X Hospital in, say, the north-east district, which is adjacent to that in which the accident has occurred, can take in such a case. He telephones accordingly and the case is removed in the ambulance to X Hospital, where it is at once taken in without loss of time and with the least expenditure of hospital and police energy.

The advantages of such a central bureau of information scarcely need demonstration; they are too obvious so far as the interests of the public are concerned. From a hospital point of view they are no less salient. Full returns of all the hospitals can be seen at the bureau, and by co-operation between the different institutions a surplus of certain cases in one hospital may be compensated by sending similar cases to another institution. In a teaching hospital where variety of cases is of some consequence such a mutual arrangement, if it can be managed without detriment to the interests of the patients and the general public, is a matter of great convenience. Further, the information bureau prevents anything in the nature of a hospital scandal due to misdirection of a badly injured patient. There is no possibility of the patient being refused admission to a hospital if he has taken care, or if his friends have taken care, to inquire at the bureau if there is a vacant bed. Mistakes are, of course, not entirely prevented by such a system, but a great deal of overlapping work is prevented and a great deal of economic energy is saved. The cost of upkeep of the bureau is comparatively small, since its working staff is small; the main items of expenditure are in postage and in telephone charges, the total of which does not amount to much. When these charges are spread over several hospitals, as would be the case where a number of institutions combined to establish such a bureau, the relative cost to each institution would be slight. Further use of the bureau can be made by the medical schools in connection with the advertising and announcing of lectures. At Berlin this work is undertaken chiefly by the State-supported post-graduate school in the Luisenplatz, which also acts to some extent as a hospital information bureau.

